# Development of a modified Cambridge Multimorbidity Score for use with SNOMED CT: an observational English primary care sentinel network study

**DOI:** 10.3399/BJGP.2022.0235

**Published:** 2023-05-03

**Authors:** Ruby SM Tsang, Mark Joy, Heather Whitaker, James P Sheppard, John Williams, Julian Sherlock, Nikhil Mayor, Bernardo Meza-Torres, Elizabeth Button, Alice J Williams, Debasish Kar, Gayathri Delanerolle, Richard McManus, FD Richard Hobbs, Simon de Lusignan

**Affiliations:** Nuffield professor of primary care health sciences, Nuffield Department of Primary Care Health Sciences, University of Oxford, Oxford.; Nuffield professor of primary care health sciences, Nuffield Department of Primary Care Health Sciences, University of Oxford, Oxford.; Public Health England, London.; Nuffield professor of primary care health sciences, Nuffield Department of Primary Care Health Sciences, University of Oxford, Oxford.; Nuffield professor of primary care health sciences, Nuffield Department of Primary Care Health Sciences, University of Oxford, Oxford.; Nuffield professor of primary care health sciences, Nuffield Department of Primary Care Health Sciences, University of Oxford, Oxford.; Royal Surrey NHS Foundation Trust, Guildford.; Nuffield professor of primary care health sciences, Nuffield Department of Primary Care Health Sciences, University of Oxford, Oxford.; Nuffield professor of primary care health sciences, Nuffield Department of Primary Care Health Sciences, University of Oxford, Oxford.; Nuffield professor of primary care health sciences, Nuffield Department of Primary Care Health Sciences, University of Oxford, Oxford.; Nuffield professor of primary care health sciences, Nuffield Department of Primary Care Health Sciences, University of Oxford, Oxford.; Nuffield professor of primary care health sciences, Nuffield Department of Primary Care Health Sciences, University of Oxford, Oxford.; Nuffield professor of primary care health sciences, Nuffield Department of Primary Care Health Sciences, University of Oxford, Oxford.; Nuffield professor of primary care health sciences, Nuffield Department of Primary Care Health Sciences, University of Oxford, Oxford.; Nuffield Department of Primary Care Health Sciences, University of Oxford, Oxford; director, Royal College of General Practitioners (RCGP) Research and Surveillance Centre, RCGP, London.

**Keywords:** general practice, medical record systems, computerised, mortality, multimorbidity, population surveillance, Systematized Nomenclature of Medicine–Clinical Terms

## Abstract

**Background:**

People with multiple health conditions are more likely to have poorer health outcomes and greater care and service needs; a reliable measure of multimorbidity would inform management strategies and resource allocation.

**Aim:**

To develop and validate a modified version of the Cambridge Multimorbidity Score in an extended age range, using clinical terms that are routinely used in electronic health records across the world (Systematized Nomenclature of Medicine — Clinical Terms, SNOMED CT).

**Design and setting:**

Observational study using diagnosis and prescriptions data from an English primary care sentinel surveillance network between 2014 and 2019.

**Method:**

In this study new variables describing 37 health conditions were curated and the associations modelled between these and 1-year mortality risk using the Cox proportional hazard model in a development dataset (*n* = 300 000). Two simplified models were then developed — a 20-condition model as per the original Cambridge Multimorbidity Score and a variable reduction model using backward elimination with Akaike information criterion as the stopping criterion. The results were compared and validated for 1-year mortality in a synchronous validation dataset (*n* = 150 000), and for 1-year and 5-year mortality in an asynchronous validation dataset (*n* = 150 000).

**Results:**

The final variable reduction model retained 21 conditions, and the conditions mostly overlapped with those in the 20-condition model. The model performed similarly to the 37- and 20-condition models, showing high discrimination and good calibration following recalibration.

**Conclusion:**

This modified version of the Cambridge Multimorbidity Score allows reliable estimation using clinical terms that can be applied internationally across multiple healthcare settings.

## INTRODUCTION

Many epidemiological analyses, including measuring the impact of disease or the effectiveness of therapies, require a single measure of comorbidity. People with multiple health conditions (‘multimorbidity’) are likely to have poorer health outcomes and require more intensive treatment and monitoring, placing significant and increasing demand across the spectrum of health services.^1^ Evaluating multimorbidity is important in allocating resources, optimising management strategies, and facilitating research. This can be achieved through composite scores that quantify the effect of specific comorbid conditions on health service use, unplanned hospital admission, and mortality.^2^^,^^3^

There have been a number of approaches to measuring comorbidity. The Charlson Comorbidity Index (CCI) is a commonly used composite morbidity score with condition weightings based on mortality.^2^ However, the management of multimorbidity has seen a paradigm shift towards a greater focus on primary care and non-hospital management of disease;^4^^–^7 the CCI, having been designed for use in secondary care and based on secondary care coding systems, is not ideal for use in primary care. Moreover, the contribution of its 12 selected comorbidities since validation in 1987 has changed, requiring the index to be re-evaluated and re-validated. Other approaches have included the number of comorbidities, although the weakness of this is the lack of weighting, which captures variation in the influence of certain conditions.

To improve on these limitations, the Cambridge Multimorbidity Score (CMMS) was developed in 2020 for use in primary care practices, using data from the Clinical Practice Research Datalink (CPRD).^8^ The CMMS used 37 conditions (and 20 in its simplified form) to predict primary care consultations, unplanned hospital admissions, and death as primary outcomes. The weighting-based outcome- specific scores of the CMMS are reported to outperform the CCI across all three primary outcomes. However, the original analysis excluded patients aged <21 years, which may limit its validity and utility in studies that include individuals outside of this age range.

**Table:** How this fits in

Multimorbidity is associated with poorer health outcomes and greater care needs in those with COVID-19, flu, and other infections. A reliable measure of multimorbidity, such as the Cambridge Multimorbidity Score (CMMS), is needed to support infectious disease epidemiology and other research based on routine data that have their key data recorded using SNOMED CT (Systematized Nomenclature of Medicine — Clinical Terms). A modified version of the CMMS was developed and validated using SNOMED CT, which is more widely used internationally than the Read terminology used for the original version and with an extended age range that the CMMS can be applied to. The unadjusted 21-condition model performed similarly to the original CMMS in predicting mortality with excellent discrimination and reasonable calibration.

The CMMS was originally developed and validated using comorbidities defined with Read clinical terminology, a thesaurus of clinical terms used to record patient findings and procedures in computerised medical records (CMR).^9^ Since April 2016 the Read terminology has not been updated. It was then retired from clinical use in English general practice in 2018 and replaced by Systematized Nomenclature of Medicine — Clinical Terms (SNOMED CT),^10^ which is used in electronic health records across the world. Potential benefits of SNOMED CT include its comprehensive nature, its capability to be machine processed, its precise collection of clinical terminology, as well as its international implementation.

During the COVID-19 pandemic it was repeatedly reported that comorbid conditions can have a detrimental impact on the severity and prognosis of COVID- 19, and individuals with underlying comorbidities are at much higher risk of severe outcomes and mortality.^11^ It is therefore imperative that surveillance and epidemiological studies account for such comorbidities. In order to support the surveillance activity of Oxford–Royal College of General Practitioners (RCGP) Research and Surveillance Centre (RSC), it is essential to have a single comorbidity index that can be used in these studies. The current study was conducted to develop and validate a modified version of the CMMS with an extended age range, which is solely based on SNOMED CT, and using routinely collected primary care data from the RSC.

## METHOD

### Data source and variables

The study used pseudonymised CMR data from the RSC sentinel network database, which is recruited to be representative of the general population.^12^ The UK has registration-based primary care in which each patient registers with a single general practice. All patients who were registered for ≥12 months before the study index date (study start date) and aged ≥16 years at the study index date for each model were included.

**Figure 1.**
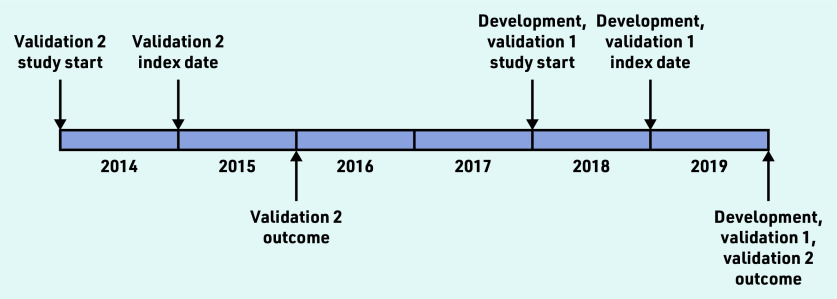
*Study index dates and study start dates for the development and validation cohorts (see main text for more detailed description).*

The cohort was split into three separate datasets (development set, validation set 1 with synchronous outcome, and validation set 2 with synchronous and asynchronous outcomes) (Figure 1) using block randomisation in the ratio of 2:1:1. To minimise the effect of random variation between practices on mortality, the cohort was separated into four subsets using the best linear unbiased estimator from a mixed-effects logistic regression with age (standardised) and sex fixed effects and a practice random effect, before block randomisation (see Supplementary Figure S1). In the current study the authors further applied similar inclusion/exclusion criteria for selecting individuals to those described in the original analysis (see Supplementary Figure S2).^8^ The authors then randomly sampled 300 000, 150 000, and 150 000 individuals from the three datasets (development set, validation set 1, and validation set 2, respectively).

The starting variables underlying the conditions used in the original development and validation were carefully curated by the authors, which was based on prior work on the epidemiology of multimorbidity in the UK,^1^^,^^13^ with the same definitions and/or prescribing before the index date applied to SNOMED CT rather than to Read v2 (see Supplementary Table S1). The exact same set of 46 starting variables was built using 66 variables within the authors’ Themes, Access, Dynamic Data Services library, and the names from the original CMMS were retained. The authors then applied the same logic and combined the anxiety and depression variables as described in Payne *et al*^8^ to yield 37 variables. Age and sex were included as covariates, with age censored at 95 years. The focus, for this study, was only on mortality as an outcome measure.

In this study variable curation is only carried out by practising clinicians with either the initial curation or quality assurance carried out by a practising GP. It involves searching for the relevant high-level concept clinical terms, called ‘supertypes’, within SNOMED CT. Clusters or individual supertypes or subtypes were excluded using a custom-built ‘helper tool’ within a secure network. The authors recorded the project the variable was curated for, the date, the data curator, and who conducted the quality assurance process and when; all recorded and retained on a secure digital environment. The clinical data curation team met weekly to ensure consistency across the variable-curation process.

The following variables were extracted: pseudonymised practice and patient identifier (ID), sex, date of birth, date of death, dates of registration and deregistration with a general practice, and the 37 conditions.

### Statistical analyses

Two time-to-mortality models were constructed using Cox proportional hazards in the development dataset. First, a model with all 37 conditions as binary indicators, with sex, age (in 10 years), and a quadratic age term included as covariates was run. Then a model was run using the 20 conditions that were considered the most important in Payne *et al*^8^ based on effect size, prevalence, and a combination of effect size and prevalence. Finally, variable reduction was conducted by entering all predictors into a model, and then using backward elimination with the Akaike information criterion (AIC)^14^ as the stopping criterion with the ‘fastbw’ function in the rms package.

Model discrimination was evaluated using pseudo-*R*^2^, Somers’ *D*, and Harrell’s *C*.^15^ *R*^2^ is a measure of explained variation in the model. Somers’ *D* quantifies the prognostic separation between observations with high and low predicted risk. Harrell’s *C* is the ratio of concordant pairs of observations to the number of comparable pairs; it estimates the concordance probability that larger predicted risks are associated with lower survival probabilities, when comparing the rankings of a pair of independent observations. Model calibration was assessed using a calibration curve, and recalibration was performed using resampling cross-validation to correct for overfitting with the ‘calibrate’ function in the rms package. Using model results from the development dataset, then performance of the models was evaluated in two validation datasets with synchronous and asynchronous outcomes (that is, 1-year follow-up in validation set 1, and 1-year and 5-year follow-ups in validation set 2 as visualised in Figure 1).

All data preparation and analyses were conducted in R (version 4.1.0),^16^ using the following packages: ggplot2 (version 0.9.1),^17^ lme4 (version 1.1-27),^18^ lubridate (version 1.7.10),^19^ randomizr (version 0.20.0),^20^ rms (version 6.2- 0),^21^ survival (version 3.2–11),^22^^,^^23^ tableone (version 0.12.0),^24^ and tidyverse (version 1.3.1).^25^

### Ethical considerations

A single comorbidity measure was required for the authors’ surveillance activity for Public Health England; its surveillance activities are now subsumed into the new UK Health Security Agency. Pseudonymised data for surveillance are extracted from volunteer general practices under Regulation 3 of the Health Service (Control of Patient Information) Regulations 2002 for health protection.

All potentially identifiable data were pseudonymised as close to source as possible and not made available to researchers; data were not extracted for patients who opted out of data sharing. All data are stored and processed at the Oxford–RCGP Clinical Informatics Digital Hub, University of Oxford. This is listed by Health Data Research UK as a trusted research environment and meets the standards of NHS Digital’s Data Security and Protection toolkit (organisation code: EE133863-MSD-NDPCHS).

## RESULTS

The three datasets were generally comparable in distribution of age, sex, number of conditions, and follow-up time (Table 1). Individuals in validation set 2 were slightly younger and healthier as a result of the earlier study index date.

**Table 1.** Descriptive statistics for the three datasets sampled from the Oxford–Royal College of General Practitioners Research and Surveillance Centre

**Characteristic**	**Development (2019) (*n* = 300 000) **	**Validation 1 (2019) (*n* = 150 000) **	**Validation 2 (2015) (*n* = 150 000)**
**Sex, male, *n* (%)**	148 672 (49.56)	74 463 (49.64)	74 527 (49.68)

**Age at index date, year**			
Mean (SD)	48.44 (19.22)	48.56 (19.39)	47.84 (19.14)
Range	16–95	16–95	16–95
65–84, *n* (%)	59 897 (19.97)	30 372 (20.25)	28 668 (19.11)
≥85, *n* (%)	9390 (3.13)	4844 (3.23)	4558 (3.04)

**Number of conditions**			
Mean (SD)	1.35 (1.85)	1.37 (1.87)	1.28 (1.78)
Range	0–15	0–15	0–14
0, *n* (%)	138 076 (46.03)	68 928 (45.95)	71 635 (47.76)
1, *n* (%)	66 053 (22.02)	32 377 (21.58)	32 400 (21.60)
≥2, *n* (%)	95 871 (31.96)	48 695 (32.46)	45 965 (30.64)

**Deaths in follow-up, *n***	3019	1433	1370/6973

**Follow-up time, days, mean**	351.5	352.3	353.2/1564

**People with complete follow-up, *n* (%)**	278 494 (92.83)	139 670 (93.11)	140 513 (93.68)/109 612 (73.07)

**Total person-years^a^**	288 722.4	144 679.6	145 041.6/642 341.4

**Mortality rate per 1000 person–years**	10.46	9.90	9.45/10.86

a *Calculated person–days then divided by 365.25. SD = standard deviation.*

The prevalence of the included 37 conditions in the development dataset is presented in Table 2. Both the rates and the rankings show similar patterns to those observed in CPRD.^8^ The top 20 conditions by prevalence and by effect size are listed in Supplementary Table S2.

**Table 2.** Prevalence of the 37 conditions in the development dataset, and the weights for the conditions included in the final model

**Condition**	**Prevalence, *n* (%) (*n* = 300 000) **	**Weight**
**Hypertension**	62 854 (20.95)	—
**Anxiety or depression**	41 744 (13.91)	0.3242
**Painful condition**	41 461 (13.82)	0.4455
**Hearing loss**	27 083 (9.03)	—
**Asthma**	22 348 (7.45)	—
**Irritable bowel syndrome**	20 671 (6.89)	−0.2037
**Diabetes**	20 232 (6.74)	0.2947
**Thyroid disorders**	18 732 (6.24)	—
**Coronary heart disease**	15 887 (5.30)	—
**Chronic kidney disease**	13 226 (4.41)	0.2137
**Diverticular disease of intestine**	10 502 (3.50)	—
**Disorder of prostate**	10 397 (3.47)	−0.1878
**Atrial fibrillation**	9105 (3.03)	0.3349
**Alcohol problems**	9064 (3.02)	0.7922
**COPD**	7542 (2.51)	0.7022
**Stroke and TIA**	7415 (2.47)	—
**Rheumatoid arthritis**	7352 (2.45)	—
**Constipation**	6311 (2.10)	0.3830
**Cancer**	5924 (1.97)	1.2026
**Peptic ulcer disease**	5071 (1.69)	—
**Chronic sinusitis**	4995 (1.67)	—
**Heart failure**	4686 (1.56)	0.5052
**Psychoactive substance misuse**	4139 (1.38)	0.4493
**Blindness and low vision**	3823 (1.27)	—
**Dementia**	3709 (1.24)	0.9380
**Psoriasis or eczema**	2794 (0.93)	—
**Epilepsy**	2580 (0.86)	0.4775
**Schizophrenia or bipolar disorder**	2402 (0.80)	0.4825
**Inflammatory bowel disease**	2371 (0.79)	—
**Chronic liver disease and viral hepatitis**	2345 (0.78)	0.6862
**Anorexia or bulimia**	2222 (0.74)	—
**Migraine**	1594 (0.53)	—
**Bronchiectasis**	1530 (0.51)	—
**Learning disability**	1290 (0.43)	0.6373
**Parkinsonism**	920 (0.31)	0.5462
**Multiple sclerosis**	853 (0.28)	0.7616
**Peripheral vascular disease**	650 (0.22)	0.3346

*COPD = chronic obstructive pulmonary disease. TIA = transient ischaemic attack.*

Discrimination of 1-year mortality using the 37-condition model were high in both validation sets 1 and 2 (Harrell’s *C* 0.92 for both models), and discrimination of 5-year mortality was only marginally worse in the validation dataset (Harrell’s *C* 0.91) (Table 3). Prediction of 1-year and 5-year mortality using the original simplified 20-condition model showed a similar pattern.

**Table 3.** Model discrimination, as assessed using pseudo-*R*^2^, Somers’ *D*, and Harrell’s *C*

**Statistic**	**37-condition model**	**20-condition model**	**Reduced model**
**Pseudo-*R*^2^**	0.153	0.152	0.153

**Somers’ *D***	0.851	0.847	0.851

**Harrell’s *C*^a^**			
Development	0.9253 (0.0022)	0.9236 (0.0022)	0.9255 (0.0021)
Validation 1	0.9200 (0.0035)	0.9184 (0.0035)	0.9206 (0.0035)
Validation 2, 1-year follow-up	0.9204 (0.0033)	0.9182 (0.0033)	0.9203 (0.0033)
Validation 2, 5-year follow-up	0.9071 (0.0016)	0.9055 (0.0016)	0.9072 (0.0016)

a *Data in brackets are standard error.*

**Figure 2.**
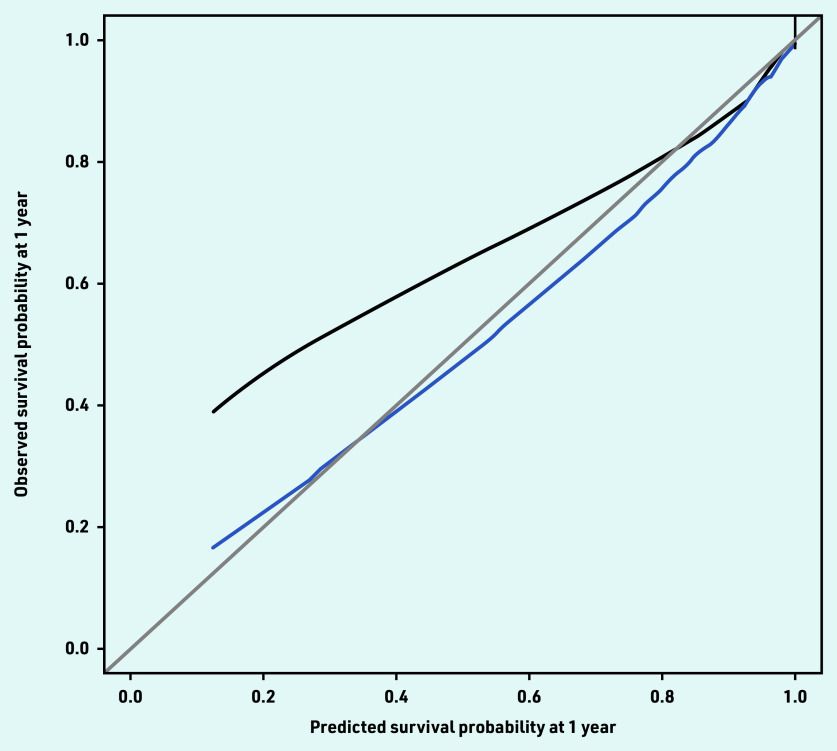
*Observed–predicted calibration curve for the 21-condition model using 2000 bootstrap samples. Mean 0.008 (standard error = 0.9). Quantile 0.01. Black = observed. Blue = optimism corrected. Grey = ideal.*

The current study’s reduced model retained 21 conditions, which partly overlapped with those in the 20-condition model (Table 4), and showed similar performance. The model had reasonable calibration, although it was found to underpredict survival at lower risks (<60%). Much of this underprediction was removed in predictions adjusted for overfitting (Figure 2).

**Table 4.** HRs (95% CIs) of the predictors from the three models^a^

**Predictor**	**37-condition model**	**20-condition model**	**Reduced model**
**Age, 10 years**	1.22 (1.02 to 1.47)	1.24 (1.03 to 1.49)	—
**[Age, 10 years]^2^**	1.05 (1.03 to 1.06)	1.05 (1.03 to 1.06)	1.06 (1.06 to 1.06)
**Sex, male**	1.33 (1.23 to 1.45)	1.29 (1.19 to 1.39)	1.34 (1.24 to 1.46)
**Cancer in the past 5 years**	3.31 (2.99 to 3.67)	3.23 (2.92 to 3.58)	3.33 (3.00 to 3.69)
**Dementia**	2.57 (2.33 to 2.84)	2.60 (2.35 to 2.87)	2.55 (2.32 to 2.82)
**Alcohol problems**	2.17 (1.84 to 2.55)	2.52 (2.18 to 2.92)	2.21 (1.88 to 2.60)
**Multiple sclerosis**	2.13 (1.32 to 3.44)	—	2.14 (1.33 to 3.46)
**Chronic liver disease and viral hepatitis**	1.98 (1.57 to 2.49)	—	1.99 (1.58 to 2.50)
**Chronic obstructive pulmonary disease**	1.96 (1.76 to 2.18)	1.97 (1.77 to 2.18)	2.02 (1.83 to 2.23)
**Learning disability**	1.88 (1.14 to 3.10)	—	1.89 (1.15 to 3.11)
**Parkinsonism**	1.71 (1.39 to 2.11)	—	1.73 (1.40 to 2.13)
**Heart failure**	1.66 (1.49 to 1.85)	1.67 (1.50 to 1.86)	1.66 (1.49 to 1.84)
**Epilepsy**	1.59 (1.25 to 2.02)	1.61 (1.27 to 2.04)	1.61 (1.27 to 2.04)
**Schizophrenia or bipolar disorder**	1.59 (1.22 to 2.06)	1.65 (1.27 to 2.13)	1.62 (1.25 to 2.10)
**Psychoactive substance misuse**	1.57 (1.20 to 2.04)	—	1.57 (1.20 to 2.04)
**Painful condition**	1.55 (1.42 to 1.68)	1.56 (1.43 to 1.69)	1.56 (1.44 to 1.69)
**Constipation**	1.47 (1.33 to 1.62)	1.51 (1.37 to 1.67)	1.47 (1.33 to 1.62)
**Peripheral vascular disease**	1.39 (1.07 to 1.81)	—	1.40 (1.08 to 1.82)
**Atrial fibrillation**	1.39 (1.27 to 1.53)	1.39 (1.26 to 1.52)	1.40 (1.27 to 1.53)
**Anxiety or depression**	1.38 (1.27 to 1.50)	1.41 (1.29 to 1.53)	1.38 (1.27 to 1.50)
**Diabetes**	1.31 (1.20 to 1.43)	1.33 (1.22 to 1.45)	1.34 (1.23 to 1.46)
**Psoriasis or eczema**	1.27 (1.03 to 1.57)	—	—
**Chronic kidney disease**	1.24 (1.14 to 1.35)	1.24 (1.14 to 1.36)	1.24 (1.14 to 1.35)
**Anorexia or bulimia**	1.22 (0.66 to 2.28)	—	—
**Peptic ulcer**	1.13 (0.98 to 1.30)	—	—
**Stroke and TIA**	1.11 (1.00 to 1.24)	1.11 (1.00 to 1.23)	–
**Bronchiectasis**	1.11 (0.87 to 1.41)	—	—
**Asthma currently treated**	1.05 (0.93 to 1.18)	1.04 (0.93 to 1.17)	—
**Hypertension**	1.04 (0.96 to 1.13)	1.04 (0.96 to 1.13)	—
**Thyroid disorders**	1.03 (0.92 to 1.14)	—	—
**Coronary heart disease**	1.00 (0.91 to 1.09)	0.99 (0.91 to 1.08)	—
**Rheumatoid arthritis**	0.98 (0.85 to 1.12)	0.98 (0.86 to 1.13)	—
**Chronic sinusitis**	1.98 (1.57 to 2.49)	—	—
**Blindness and low vision**	0.96 (0.84 to 1.11)	—	—
**Hearing loss**	0.92 (0.85 to 1.00)	0.92 (0.85 to 1.00)	—
**Diverticular disease of intestine**	0.92 (0.82 to 1.02)	—	—
**Disorder of prostate**	0.83 (0.74 to 0.93)	—	0.83 (0.74 to 0.93)
**Irritable bowel syndrome**	0.83 (0.71 to 0.95)	0.81 (0.70 to 0.94)	0.82 (0.71 to 0.94)
**Inflammatory bowel disease**	0.65 (0.43 to 0.97)	—	—
**Migraine**	0.59 (0.25 to 1.42)	—	—

a *A blank cell indicates that the risk factor concerned was not included in the relevant model. HR = hazard ratio. TIA = transient ischaemic attack.*

## DISCUSSION

### Summary

In this study, a modified version of the CMMS was developed and validated for use with a wider age range and using SNOMED CT. The reduced 21-condition model performed similarly to both the full model and the original 20-condition model in predicting mortality with excellent discrimination and reasonable calibration. The performance of the model was also similar to that of the original CMMS. The authors have opted to use the unadjusted 21-condition model as this will maximise its use in studies of different designs, where researchers can apply their own adjustments for age and sex. The authors plan to use this multimorbidity score in their epidemiological studies (including COVID-19 studies), and to make this available to the wider international SNOMED CT community.

### Strengths and limitations

The study used a large, up-to-date, nationally representative cohort, which included all patients aged ≥16 years, and the results were validated using both synchronous and asynchronous datasets. As this study arose from the need for a single comorbidity measure to support the authors’ surveillance activity during the pandemic the authors sampled the development and validation datasets only from their cohort and therefore the index was not validated in other independent samples. The analysis was based on SNOMED CT, which is now used across English general practice as well as internationally. The authors believe the results are generalisable to other cohorts and potentially other countries that use similarly coded primary care data. A full list of clinical terms that make up the variables are provided in Supplementary Appendix S1. The authors’ logic model and weightings, for others to use in other databases, are provided in Supplementary Appendix S2.

CMMS weights were only derived for mortality and not unplanned hospital admissions or primary care consultations. Mortality tends to be the most commonly used outcome in the development of comorbidity indices in the literature,^26^ and it was felt that having only one set of weights would allow it to be easier to apply to, and interpret in, different datasets.

The list of conditions used in this study were exactly as included in the original development and validation of the CMMS by Payne *et al*,^8^ which was based on earlier literature on multimorbidity in primary care.^1^^,^^13^ These studies did not include other common conditions that might be expected to be included in other multimorbidity indices or that are highly clinically relevant (for example, obesity).

### Comparison with existing literature

A number of comorbidity indices and adaptations have been developed in administrative data studies, which are either solely diagnosis based or solely medication based.^26^ The current score uses a different approach that combines information from clinical terms as well as prescriptions, and additionally includes a 12-month timeframe in the definition of certain conditions. This allows the severity and/or recency of some conditions (for example, constipation and cancer) to be taken into account in the calculation of the score.

The current study retained a slightly different set of conditions in the reduced model to that in Payne *et al*.^8^ As variable reduction in the current model was based on AIC rather than the combination of effect size and prevalence, the modified score included some less prevalent conditions that are strongly associated with mortality such as multiple sclerosis and learning disability. The differences in included conditions and weightings between the current model and Payne *et al*’s^8^ may also be partly explained by age group differences in multimorbidity patterns as the current study used a lower age cut-off of 16 years. Earlier research has shown that, although multimorbidity in later life tends to involve multiple ‘concordant’ conditions (typically vascular and metabolic conditions), multimorbidity in earlier adulthood generally involves a mix of physical and mental conditions.^27^

### Implications for research and practice

With an ageing population, multimorbidity is increasingly prevalent, particularly in developed countries. Multimorbidity is known to be associated with higher healthcare use, higher healthcare costs, greater mortality, as well as higher risk of active patient safety incidents (for example, adverse drug events) or precursors of safety incidents (for example, medication non-adherence).^28^ Some earlier studies also suggest particular clusters of multimorbidity, for example, those that involve mental disorders, may be associated with poorer outcomes,^28^ and this is also reflected in the modified version of CMMS where a number of mental conditions and substance use disorders are included. A better understanding of multimorbidity and the specific conditions or clusters that lead to poorer outcomes would inform healthcare coordination and delivery, and the development of interventions to improve outcomes in the subgroups of patients at highest risk.

As this version of the CMMS was developed based on a population-based cohort, its relevance and generalisability to specialised settings, for example, a mental health service where the patients’ demographic and clinical characteristics may be quite different, remains unclear. Future research should explore whether such a measure is useful in guiding care decision making and better organisation of health services in a range of settings and different subgroups of the population.

In conclusion, in this study the development and validation of a modified version of the CMMS for predicting mortality is described. The inclusion of a wider age range may improve the generalisability of the score over the original. As it is based on SNOMED CT rather than Read codes, it is applicable to today’s English general practice data and this should also increase its applicability in other contexts. Future research should investigate whether a measure of multimorbidity may be helpful in informing healthcare coordination and delivery, especially across a range of different healthcare settings.

## Data

The Oxford–RCGP RSC dataset can be accessed by researchers; approval is on a project-by-project basis (https://orchid.phc.ox.ac.uk/index.php/rcgp-rsc). Ethical approval by an NHS Research Ethics Committee/other appropriate approval is needed before any data release. Researchers wishing to directly analyse patient-level pseudonymised data will be required to complete information governance training and work on the data from the secure servers at the University of Oxford. Patient-level data cannot be taken out of the secure network.
